# A multi-centre, double-blind, randomized, placebo-controlled trial to evaluate the effectiveness and safety of ramelteon for the prevention of postoperative delirium in elderly cancer patients: a study protocol for JORTC-PON2/J-SUPPORT2103/NCCH2103

**DOI:** 10.1093/jjco/hyad061

**Published:** 2023-06-20

**Authors:** Ryoichi Sadahiro, Kotaro Hatta, Takuhiro Yamaguchi, Enokido Masanori, Yoshinobu Matsuda, Asao Ogawa, Yusei Iwata, Akihiro Tokoro, Rika Nakahara, Takatoshi Hirayama, Yuko Yanai, Yuko Ogawa, Ayako Kayano, Keisuke Ariyoshi, Shunsuke Oyamada, Yosuke Uchitomi, Tatsuo Akechi, Noboru Yamamoto, Natsuko Okita, Eiko Yorikane, Kazuaki Shimada, Tetsuya Furukawa, Hironobu Hashimoto, Makoto Maeda, Tetsufumi Sato, Asuko Sekimoto, Chiyuki Sasaki, Eiko Saito, Yasuhito Uezono, Hiromichi Matsuoka

**Affiliations:** Department of Psycho-Oncology, National Cancer Center Hospital, National Cancer Center Japan, Tokyo, Japan; Department of Psychiatry, Juntendo University Nerima Hospital, Tokyo, Japan; Division of Biostatistics, Tohoku University Graduate School of Medicine, Miyagi, Japan; Department of Psycho-Oncology, National Cancer Center East, National Cancer Center Japan, Chiba, Japan; Department of Psychosomatic Internal Medicine, National Hospital Organization Kinki-Chuo Chest Medical Center, Osaka, Japan; Department of Psycho-Oncology, National Cancer Center East, National Cancer Center Japan, Chiba, Japan; Department of Psycho-Oncology, National Cancer Center East, National Cancer Center Japan, Chiba, Japan; Department of Psychosomatic Internal Medicine, National Hospital Organization Kinki-Chuo Chest Medical Center, Osaka, Japan; Department of Psycho-Oncology, National Cancer Center Hospital, National Cancer Center Japan, Tokyo, Japan; Department of Psycho-Oncology, National Cancer Center Hospital, National Cancer Center Japan, Tokyo, Japan; Department of Psycho-Oncology, National Cancer Center Hospital, National Cancer Center Japan, Tokyo, Japan; Department of Psycho-Oncology, National Cancer Center Hospital, National Cancer Center Japan, Tokyo, Japan; Department of Psycho-Oncology, National Cancer Center Hospital, National Cancer Center Japan, Tokyo, Japan; JORTC Data Centre, NPO, Tokyo, Japan; JORTC Data Centre, NPO, Tokyo, Japan; Division of Survivorship Research, Institute for Cancer Control, National Cancer Center Japan, Tokyo, Japan; Department of Psychiatry and Cognitive-Behavioral Medicine, Nagoya City University Graduate School of Medical Sciences, Nagoya, Japan; Department of Experimental Therapeutics, National Cancer Center Hospital, Tokyo, Japan; Clinical Trial Support Office, National Cancer Center Hospital, Tokyo, Japan; Clinical Trial Support Office, National Cancer Center Hospital, Tokyo, Japan; National Cancer Center Hospital, National Cancer Center Japan, Tokyo, Japan; Department of Pharmacy, National Cancer Center Hospital, National Cancer Center Japan, Tokyo, Japan; Department of Pharmacy, National Cancer Center Hospital, National Cancer Center Japan, Tokyo, Japan; Department of Pharmacy, National Cancer Center Hospital, National Cancer Center Japan, Tokyo, Japan; Department of Anesthesia and Intensive Care, National Cancer Center Japan, Tokyo, Japan; Department of Nursing, National Cancer Center Hospital, National Cancer Center Japan, Tokyo, Japan; Department of Nursing, National Cancer Center Hospital, National Cancer Center Japan, Tokyo, Japan; Institute for Global Health Policy Research, Bureau of International Health Cooperation, National Center for Global Health and Medicine, Tokyo, Japan; Department of Pain Control Research, Jikei University School of Medicine, Tokyo, Japan; Department of Psycho-Oncology, National Cancer Center Hospital, National Cancer Center Japan, Tokyo, Japan

**Keywords:** ramelteon, delirium, randomized controlled trial, cancer

## Abstract

Postoperative delirium is an important issue in cancer patients, affecting surgical outcomes and the quality of life. Ramelteon is a melatonin receptor agonist with high affinity for MT1 and MT2 receptors. Clinical trials and observational studies in Japan, including in surgical cancer patients, have shown efficacy of ramelteon in delirium prevention, with no serious safety concerns. However, clinical trials from the USA have reported conflicting results. A Japanese phase II study investigated the efficacy and safety of ramelteon for delirium prevention following gastrectomy in patients aged ≥75 years, with findings suggesting the feasibility of a phase III trial. The aim of this multi-centre, double-blind, randomized placebo-controlled phase III trial is to evaluate the effectiveness and safety of oral ramelteon for postoperative delirium prevention in cancer patients aged ≥65 years as advanced medical care. The trial protocol is described here.

## Teaser

This paper describes the protocol of a multi-centre, double-blind, randomized placebo-controlled trial evaluating the effectiveness and safety of oral ramelteon for postoperative delirium prevention in cancer patients aged ≥65 years.

## Introduction

Delirium is an acute disturbance of consciousness that occurs in response to surgical invasion in about half of all hospitalized older people (1). It causes cognitive dysfunction including impaired attention, hallucinations, delusions and other thought disturbances, as well as impaired communication, which can cause distress to the patient, their family and medical staff; even if resolved, it can result in reduced independence, increased mortality and cognitive decline (2). It is also associated with a substantial cost burden, with a recent systematic review reporting per-patient costs of delirium ranging between US$806 and US$24 509 (3). Delirium is an important issue in oncology and palliative medicine, reducing surgical outcomes and quality of life. According to previous research, delirium (54.7%) was cited more than pain (46.9%) as a symptom that palliative physicians struggled to manage during supportive and palliative care (4). The incidence of postoperative delirium is substantial. We previously reported that delirium occurred in 32% of patients after surgical resection of cancer (5). Furthermore, in line with the aging population, the number of cancer resections in older people is increasing, with older people being at a high risk of postoperative delirium. Nevertheless, despite its burden and impact, no drug to date has been shown to be both safe and effective in preventing delirium, and as a result, there is no established method for its prevention (6).

Previous studies have demonstrated that excessive inflammatory response and elevated dopamine levels in peripheral blood are involved in postoperative delirium (7). Melatonin has the effect of reducing the inflammatory response to invasion and suppressing dopamine release (8,9). As insomnia appears to be a modifiable risk factor for delirium, it is plausible that melatonin may prevent delirium by improving sleep (10). The development of novel melatonin-based prophylaxis against delirium has been proposed as a potential approach but has not yet been initiated in Japan or overseas (11). Additionally, the discontinuation of medications that might increase the risk of delirium is recommended as part of combined interventions (12). It has been suggested that ramelteon, a melatonin receptor agonist with high affinity for MT1 and MT2 receptors (13), may be effective in preventing delirium through allowing the discontinuation of benzodiazepines and non-benzodiazepines, which are risk factors for delirium (14).

Ramelteon was approved in Japan in 2010 for treating insomnia (15). Observational studies in Japan have reported the consistent efficacy and safety of ramelteon in preventing postoperative delirium (14,16–18). A phase II study demonstrated the efficacy and safety of ramelteon for the prevention of delirium after gastrectomy in gastric cancer patients aged ≥75 years (19). These findings suggest the feasibility of a phase III trial.

A recent network meta-analysis of pharmacological interventions for delirium reported ramelteon to be the most promising drug for the prevention of delirium (20). The dual orexin receptor antagonist suvorexant appears to have similar efficacy to ramelteon (20). However, it has a mild risk of dependence and is listed as a Schedule IV drug by the Food and Drug Administration (21), raising concerns about its use in prophylaxis. Ramelteon has been shown to be effective and safe for the treatment of delirium associated with acute illness in randomized controlled trials conducted in Japan (22,23). Two randomized controlled studies in the USA investigated the efficacy of ramelteon in preventing delirium in patients undergoing orthopaedic surgery (24) and pulmonary thromboendarterectomy (25). Both studies reported that the drug was not effective in preventing postoperative delirium, in contrast to Japanese studies suggesting efficacy (14,16–19).

In summary, the efficacy of the melatonin receptor agonist ramelteon for treating delirium has not yet been conclusively established. We hypothesize that ramelteon may offer an effective and safe new standard of care for the prevention of postoperative delirium after cancer resection, and we plan to conduct a phase III trial to resolve this off-label use under Japan’s Advanced Medical Care System.

## Protocol digest of JORTC-PON2/J-SUPPORT2103/NCCH2103

### Trial registration and institutional review board approval

The study protocol has received ethical approval from the Clinical Research Review Committee of the National Cancer Center Hospital (CRB3180008). The trial is registered in the jRCT clinical trials registry: jRCTs031210673. The physician approved as the researcher by the Clinical Research Review Committee of the National Cancer Center Hospital (CRB3180008) will obtain written consent from each study participant using an informed consent form.

### Advanced medical care system

In Japan, ramelteon has already received manufacturing and marketing approval under the Pharmaceutical Affairs Law for the treatment of difficulty falling asleep in insomnia but not for the prevention of delirium. To investigate off-label use under the National Health Insurance system, this clinical trial has been approved by the Ministry of Health, Labour and Welfare's ‘Advanced Medical Care System’ (26). If a certain level of results is achieved under the Advanced Medical Care System, it is then possible to expand insurance coverage to include off-label use of ramelteon for treating delirium as an unapproved drug subject to the Expedited Practical Application Scheme for Unapproved Drugs.

### Aims

This study aims to evaluate the effectiveness and safety of oral ramelteon for the prevention of postoperative delirium in elderly cancer patients in Japan.

### Study design

**Figure 1:**
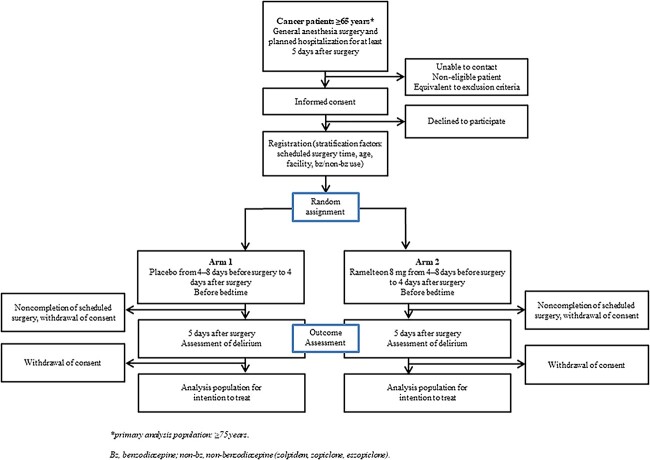
Flowchart of the study procedure.

The study is a multi-centre, double-blind, placebo-controlled, randomized controlled trial. The flowchart of the study procedure is shown in Fig. 1.

### Study setting and patients

Cancer patients aged ≥65 who are scheduled to undergo surgery under general anaesthesia and hospitalization for at least 5 days after surgery at the National Cancer Center Hospital (Tokyo), East Hospital (Chiba) or Kinki Central Respiratory Center (Osaka) will be included. The study will take place from April 2022 to March 2024, with a 12-week follow-up period.

### Eligibility criteria

Patients who meet all the following criteria are eligible:

1. Patients with pathologically confirmed malignancy and patients with a strong clinical suspicion of malignancy.
2. Patients aged ≥65 years at the time of registration.
3. Patients scheduled for surgery under general anaesthesia and hospitalization for at least 5 days postoperatively.
4. Patients eligible for oral or nasogastric tube medication preoperatively and who are expected to resume oral or nasogastric tube medication within 2 days postoperatively.
5. Patients whose written consent to participate in the study has been obtained from them. Patients who have given their verbal and written consent in the presence of a witness (other than those engaged in the clinical research) and signed by the witness.

### Exclusion criteria

Patients in whom any one of the following applies are excluded:

1. Patients with a Diagnostic and Statistical Manual of Mental Disorders-5 (DSM-5) (27) diagnosis of delirium at the time of enrolment.
2. Patients with a history of allergy to ramelteon.
3. Patients with severe hepatic dysfunction defined as aspartate transaminase >90 U/L, alanine transaminase (ALT) >126 U/L (men), ALT >69 U/L (women) and total bilirubin >2.25 mg/dl.
4. Patients who were participating in a clinical trial or clinical study with a new drug within 4 weeks of enrolment, including the date of enrolment (patients who have completed protocol treatment and are in the observation period are excluded).
5. Patients taking ramelteon within 2 weeks of registration, including the date of registration, and fluvoxamine, with which there is a known interaction (28).
6. Patients who have taken an orexin receptor antagonist (suvorexant or lemborexant) within 2 weeks of enrolment, including the date of enrolment.
7. Patients with a history of epilepsy, Parkinson’s disease or dementia with Lewy bodies.
8. Patients with a history of lactose intolerance.
9. Patients whose planned resection includes central nervous system and intracranial areas.
10. Patients with drug or alcohol use disorders within 5 years of enrolment, including the date of enrolment; patients who consume an average of 60 g of alcohol daily (e.g. 3 g of sake, three medium bottles of beer and five glasses of wine) or more; and patients with alcohol-related diseases (e.g. alcoholic liver disease).
11. Patients who have previously participated in this study.
12. Patients with moderate or severe dementia (Mini-Mental State Examination Japanese Version score < 21) (29).
13. Patients with severe visual, hearing or reading impairments.
14. Patients deemed unsuitable by the principal investigator or sub-investigator.

### Endpoints

The primary efficacy endpoint is the proportion of patients aged ≥75 years with delirium in the first 5 days after surgery as diagnosed by a trained psycho-oncologist using the DSM-5 (27). The secondary efficacy endpoints are as follows:

1. Delirium in the first 5 days after surgery as diagnosed by a psycho-oncologist using the DSM-5. This will be verified only if a significant effect is found in the primary analysis population. If a significant effect is not found in the primary analysis population, this will be analysed as an exploratory endpoint only.
2. The remaining secondary endpoints will be investigated in all age groups and using stratified analyses (stratified into patients aged ≥75 years).
3. The proportion of delirium in the first 5 days after surgery as diagnosed by psycho-oncologists using the DSM-5 (excluding delirium within 2 hours after surgery).
4. Proportion of delirium in the first 5 days after surgery as assessed by nurses using the Nursing Delirium Screening Scale (Nu-DESC) (30).
5. Delirium severity at 5 days after surgery as assessed using the Japanese version of the Delirium Rating Scale (DRS)-R-98 (31,32) and Nu-DESC.
6. The proportion of cases of severe delirium in the first 5 days after surgery diagnosed by DSM-5 and DRS-R-98.
7. Days from diagnosis to resolution of delirium according to DSM-5.
8. Direct medical costs from post-surgery to discharge.
9. Postoperative hospital stay.
10. The proportion of antipsychotic use in the first 5 days after surgery.
11. The proportion of patients stopping or withdrawing from benzodiazepines and non-benzodiazepines on the day prior to surgery.
12. The proportion of falls, self-removal of any tubes including drip infusions and self-extubation and physical restraint within 5 days of surgery.
13. Serum C-reactive protein level on the morning of the first postoperative day.
14. Percentage of postoperative complications [Clavien–Dindo (CD) category grade ≥ 2] in the first 5 days after surgery.
15. The proportion of postoperative complications (CD grade ≥ 2) within 90 days of surgery.
16. Survival within 30 days after surgery.
17. Survival up to 90 days after surgery.
18. Recurrence-free survival within 90 days after surgery.
19. Patient satisfaction with study participation and subjective symptoms on postoperative day 5 (Edmonton Symptom Rating System, modified Japanese version) (33).
20. Percentage of patients who cannot be discharged home.
21. Proportion of delirium in the first 5 days after surgery diagnosed by a psycho-oncologist using the DSM-5, with a Pittsburgh Sleep Quality Index (PSQI) (34) of ≥6 and a PSQI of ≤5 at enrolment.

### Safety assessments

Safety assessments include adverse events and adverse reactions during the treatment period until postoperative day 5. Investigation items for safety assessment are somnolence, floating dizziness, malaise, headache, constipation, nausea and urticaria. Adverse events related to surgery will not be included in this study. The investigator or sub-investigator will consult with the surgeon at each site to determine whether an adverse event is surgery related. Common Terminology Criteria for Adverse Events v5.0 grade 2 or higher will be treated as an adverse event.

### Randomization

The study will employ an electronic data capture (EDC) system for case enrolment and random assignment of treatment groups using the minimization method. Blinding of the study is conducted based on an allocation chart prepared by the study drug allocation manager, with only the drug number notified by the EDC system. Stratification factors include age ≥ 75 years at enrolment, highly invasive surgery scheduled for ≥6 hours, benzodiazepine and non-benzodiazepine use and the site of enrolment.

### Treatment methods

A flowchart of the study procedure is shown in Fig. 1. The study schedule is shown in Table 1. The test drug comprises Swedish orange capsules filled with ramelteon 8 mg formulation and an identical control capsule containing a lactose tablet of the same specification (same size and weight but ramelteon-free). One capsule of the study drug (test or control) will be administered orally or via a nasogastric tube once daily before bedtime during the medication period (double-blind). As a co-intervention, a combined intervention for the prevention of delirium will be implemented in all patients, according to the National Institute for Health and Clinical Excellence Guideline ‘Delirium: prevention, diagnosis and management’, e.g. patients are provided clinically appropriate care if they have cognitive impairment, disorientation, dehydration, constipation, hypoxia, infection, immobility, pain, benzodiazepine and/or anti-benzodiazepine prescription, poor nutrition, sensory impairment and insomnia (12).

**Table 1:** Study schedule

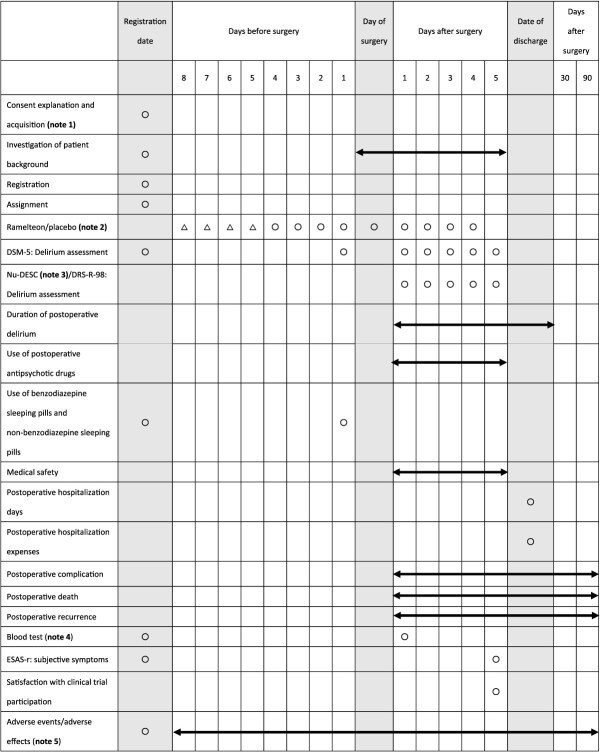

Note 1: If the patient comes to the hospital after consent has been obtained and before preoperative admission, consent will not be obtained again.

Note 2: If the duration of administration can be secured, protocol treatment will start earlier during a period denoted by triangles.

Note 3: The Nursing Delirium Screening Scale will only be administered at the National Cancer Center Hospital.

Note 4: Details of the blood tests are as follows:

1. Total bilirubin, aspartate transaminase (AST) and alanine transaminase (ALT) at the date of registration are adopted from blood test results performed in clinical practice within 8 weeks retrospectively from the date of registration.

2. Adopt clinically performed blood test results for total bilirubin, AST, ALT and C-reactive protein on the first postoperative day.

Note 5: Adverse effects such as somnolence, floating dizziness, malaise, headache, constipation, nausea and urticaria should be investigated.

ALT, alanine transaminase; AST, aspartate transaminase; DRS, Delirium Rating Scale; DSM, Diagnostic and Statistical Manual of Mental Disorders; ESAS-r; Edmonton Symptom Assessment System Revised Japanese Version; Nu-DESC, Nursing Delirium Screening Scale

### Follow-up

The planned follow-up period is 12 weeks.

### Sample size

The proportion of delirium in patients aged ≥75 years is assumed to be 24% in the ramelteon group and 36% in the control group. With a two-sided alpha error of 0.05 and power of 0.90, the required sample size is calculated at 610 patients. We therefore plan to enrol 678 patients aged ≥75 years (339 per group) to allow for a 10% dropout. The proportion of delirium in patients aged ≥65 years is assumed to be 22% in the ramelteon group and 33% in the control group. With a two-sided alpha error of 0.05 and power of 0.90, the required sample size is calculated at 690 patients. Therefore, 766 patients (383 per group) would be required to allow for 10% dropout. We thus plan to enrol 88 patients aged 65–74 years in addition to the 678 patients aged ≥75 years. The study will continue to enrol both age groups until the planned enrolment of 88 patients in the 65- to 74-year-old group and 678 patients in the ≥75-year-old group is met.

### Statistical analysis

We will calculate point estimates and 95% confidence intervals for the proportion of patients in each group aged ≥75 years with delirium in the 5 days after surgery as diagnosed by a psycho-oncologist using the DSM-5 and compare groups using the Cochran–Mantel–Haenszel test adjusted for stratification factors. The significance level is set at 5% (two-sided). If a statistically significant between-group difference is found for age ≥ 75, a similar comparison will be made for those aged ≥65 without adjustment for statistical multiplicity. If the superiority of the ramelteon group is confirmed, ramelteon treatment will be considered a useful prophylaxis.

### Interim analysis and monitoring

Because of the small differences between groups to be detected, no interim analysis will be performed.

### Participating institutions

The National Cancer Center Hospital (Tokyo), The National Cancer Center East Hospital (Chiba) and Kinki Central Respiratory Center (Osaka).

## Notes

Trial registration: jRCTs031210673
